# Acceleration of CRISPR/Cas9-Mediated Editing at Multiple Sites in the *Saccharomyces cerevisiae* Genome

**DOI:** 10.3390/mps6020039

**Published:** 2023-04-04

**Authors:** Alexey D. Karpukhin, Fanis A. Sabirzyanov, Vsevolod A. Serebrianyi

**Affiliations:** Ajinomoto-Genetika Research Institute, 1st Dorozhny Proezd, b.1-1, Moscow 117545, Russia; fanis_sabirzyanov@agri.ru (F.A.S.); vsevolod_serebrianyi@agri.ru (V.A.S.)

**Keywords:** CRISPR/Cas9, *Saccharomyces cerevisiae*, simultaneous multiple modifications, electroporation

## Abstract

The application of the CRISPR/Cas9-based genome editing technique to the yeast *Saccharomyces cerevisiae* has made it possible to simultaneously modify several sites, particularly to integrate several expression cassettes. The existing methods provide high efficiency for such modifications; however, common protocols include several preparatory steps, namely, the construction of an intermediate Cas9-expressing strain, the assembly of a plasmid bearing several single guide RNA (sgRNA) expression cassettes, and the surrounding integrated DNA fragments with long flanks for recombination with target loci. Since these preparatory steps are time consuming and may not be desirable in some types of experiments, we explored the possibility of multiple integration without these steps. We have demonstrated that it is possible to skip them simultaneously and integrate up to three expression cassettes into separate sites by transforming the recipient strain with the Cas9 expression plasmid, three differently marked sgRNA plasmids, and three donor DNAs flanked with short (70 bp) arms for recombination. This finding increases the flexibility of choosing the optimal experimental design for multiple editing of the genome of *S. cerevisiae* and can significantly accelerate such experiments.

## 1. Introduction

Currently, the CRISPR/Cas9 system is successfully used to edit the genome of the yeast *Saccharomyces cerevisiae*. In the most common version, the system consists of two components: the Cas9 protein and a single guide RNA (sgRNA). These components form a ribonucleoprotein complex that introduces a double-strand break (DSB) in the genome at the site determined by the sequence of the sgRNA (for a review, see [1]). DSBs can be repaired via two mechanisms: nonhomologous end joining (NHEJ) and homology-directed repair (HR). DNA repair via HR requires a DNA template that shares sequence similarity with the damaged DNA [2]. This mechanism is used for the integration of DNA fragments into the target site. For this purpose, the DNA fragment intended for integration is flanked by sequences homologous to the regions adjacent to the DSB. The resulting construct is often referred to as the donor DNA (dDNA).

Cas9 and sgRNA can be introduced into cells in the form of a ribonucleoprotein complex or in the form of expression cassettes encoding these components. Due to the ability of *S. cerevisiae* to carry various plasmids, the introduction of plasmids bearing Cas9 and sgRNA expression cassettes is the simplest and most convenient design for CRISPR/Cas9-based experiments in this organism. Components can be cloned in one plasmid vector or on two separate plasmids. In addition, it has recently been demonstrated that sgRNA expression cassettes can be introduced into *S. cerevisiae* as linear DNA fragments, and a method has been developed to rapidly construct such fragments [3]. This discovery greatly accelerates genome editing experiments, but its application is limited to experiments in which the presence of a selectable marker in dDNA is acceptable. For introduction of plasmids with CRISPR/Cas9 components and dDNA fragment(s) into yeast cells, both lithium acetate (LiAc) transformation and electroporation have been used [4,5,6,7].

**Figure 1:**
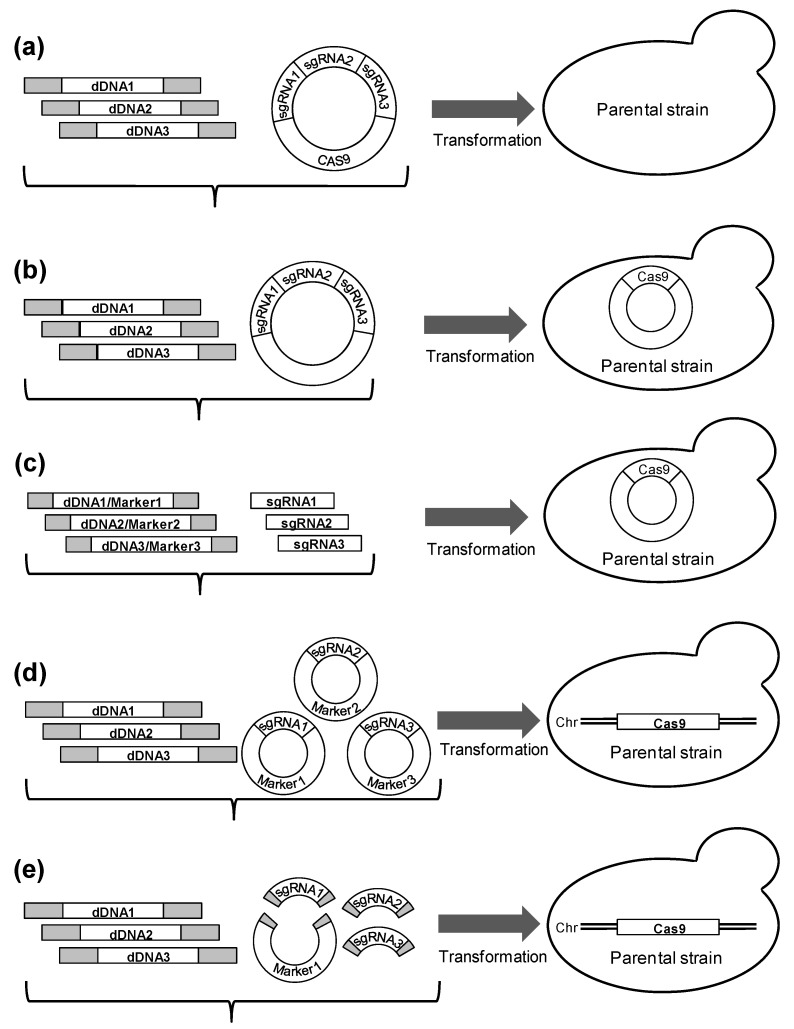
Several experimental designs for CRISPR/Cas9-mediated multiple integration. dDNA, donor DNA; sgRNA, single guide RNA. (**a**) Transformation of a recipient strain with a single plasmid bearing all sgRNA cassettes and Cas9 cassette [8,9]; (**b**,**c**) Construction of an intermediate strain bearing the Cas9 plasmid and transformation of this strain with a plasmid containing three sgRNA expression cassettes [5,7] (**b**), or with the linear DNA fragments encoding sgRNA expression cassettes [10] (**c**); (**d**) Co-transformation of the strain constitutively expressing Cas9 with three separate plasmids containing sgRNA expression cassettes and different selective markers [6]; (**e**) Co-transformation of the strain constitutively expressing Cas9 with a linearized vector and three linear sgRNA expression cassettes resulting in assembling of sgRNA plasmids in cells due to gap repair [6].

The use of the CRISPR/Cas9 system for *S. cerevisiae* has made it possible to simultaneously modify several sites. Using various protocols, simultaneous inactivation of three genes [8,9], deletion of three long chromosomal regions [10], and simultaneous integration of expression cassettes into three sites were demonstrated [5,6,7]. Various experimental designs have been used for simultaneous integration into several sites: the cloning of sgRNAs and Cas9 expression cassettes into a single vector [8,9]; construction of an intermediate strain bearing the Cas9 plasmid and transformation of this strain with a plasmid containing three sgRNA expression cassettes [5,7] or three linear DNA fragments encoding sgRNA expression cassettes [10]; co-transformation of the strain constitutively expressing Cas9 with three separate plasmids containing sgRNA expression cassettes and different selective markers [6]; and co-transformation of the strain constitutively expressing Cas9 with a linearized vector and three linear sgRNA expression cassettes resulting in assembly of sgRNA plasmids in cells due to gap repair [6] (Figure 1). The listed methods include one or two preparatory steps, namely, the creation of an intermediate strain bearing the Cas9 expression cassette and assembly of the plasmid carrying gRNA expression cassettes or gRNA expression cassettes and Cas9 expression cassette. These steps slow down the editing process and reduce the flexibility in designing experiments. Moreover, the creation of an intermediate strain is undesirable in the case of heavily modified strains, since the loss of strain properties can occur during the introduction of the Cas9 expression cassette.

To the best of our knowledge, for the integration of multiple expression cassettes, homologous arms with a length of approximately 0.5 kb are usually used [5,6]. Alternatively, the arms may not be covalently linked to the cassettes but overlap with their ends by approximately 50 bp [7]. However, the minimal length of the flanks sufficient to provide integration of the DNA fragment via HR is approximately 30 bp [11]. Such short sequences can be included in oligonucleotides and added to DNA fragments by PCR. This approach is widely used for the introduction of various modifications into the genome of *S. cerevisiae*, such as gene deletions and integration of DNA fragments [12]. For CRISPR/Cas9-mediated integration, it has also been demonstrated that short homologous arms (50–60 bp length) provide almost 100% efficiency of integration into one target site [5,8] and 83–100% efficiency of triple deletion of chromosomal regions [10]. Fifty-base-pair homologous arms were also used for the integration of short DNA fragments (for gene inactivation) into two or three pairs of homologous genes in the diploid strain. This experiment was also successful but with lower targeting efficiency (43% and 19%, respectively) [8]. Thus, long arms are not strictly necessary, at least in experiments of certain design.

**Figure 2:**
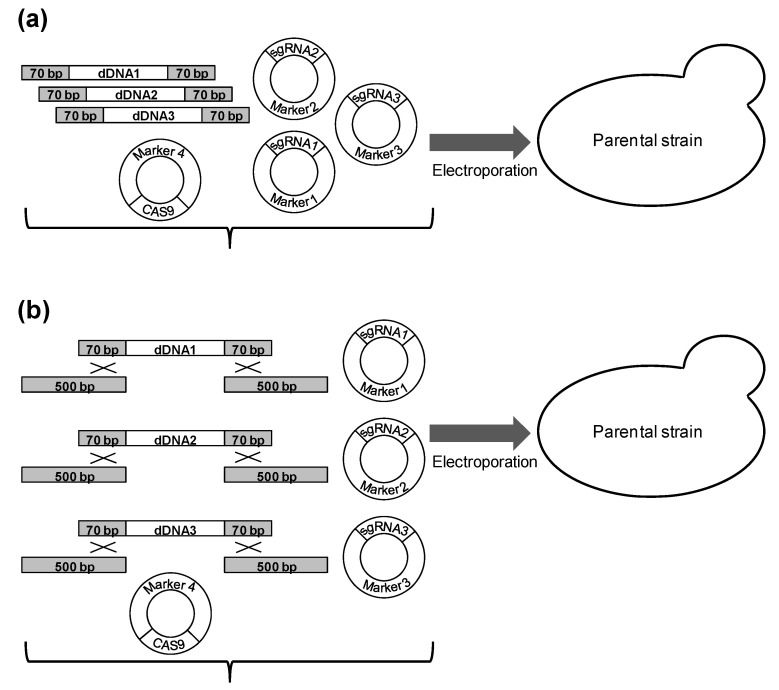
Proposed schemes for triple integration. (**a**) Transformation of the recipient strain with a plasmid expressing Cas9, three plasmids that each carrying one sgRNA expression cassette and an individual selective marker, and three dDNAs containing short (70 bp) flanks for recombination with the target locus. (**b**) The same as (**a**) but 0.5 kb arms for recombination with target loci that overlapped with integrated DNAs are added.

In summary, there are three preparatory stages that slow experiments for CRISPR/Cas9-mediated genome editing at multiple sites: (i) the assembly of a plasmid bearing several sgRNA expression cassettes, (ii) the addition of long homologous arms to the dDNA fragment, and (iii) the construction of an intermediate Cas9-expressing strain. The common protocols use all these steps or only some of them. The aim of our study was to evaluate how necessary these steps are for simultaneous CRISPR/Cas9-mediated editing of several sites and whether it is possible to exclude all of them at the same time. We have demonstrated that the introduction of a plasmid harboring the Cas9 expression cassette into a recipient strain simultaneously with three differently marked sgRNA plasmids and corresponding dDNAs only slightly reduced the proportion of transformants with triple integration, but significantly reduced the yield of transformants compared to the transformation of a strain that already carries the Cas9 plasmid. We overcame the low yield of transformants by applying electroporation instead of the standard lithium acetate method. We have also demonstrated that integration efficiency decreased significantly when the length of homologous arms was reduced from 0.5 kb to 70 bp, but it remained sufficient for integration of two fragments (with a frequency of 45%) and even three fragments (with a frequency of 6%). Thus, for triple integration, it is possible to skip all three preparatory steps at the same time and transform the recipient strain with a plasmid expressing Cas9, three plasmids, each of which carries one sgRNA expression cassette and an individual selective marker, and three dDNAs fragments containing short (70 bp) flanks for recombination with the target locus (Figure 2a). The efficiency of integration of fragments with short arms can be increased by the addition of long DNA fragments homologous to flanks of the integration sites and not linked to but overlapping with the ends of fragments subjected to integration, an approach well known in yeast molecular biology (Figure 2b). This approach increased the proportion of correct integration of the three fragments up to 24%.

## 2. Materials and Methods

### 2.1. Strains and Media

The *S. cerevisiae* haploid strains BY4742 (S288C background, *MAT* alpha *his3Δ1 leu2Δ0 lys2Δ0 ura3Δ0*), BY4742/GFP_II-1 (GFP expressing cassette in site II-1), BY4742/GFP_IX-1 (GFP expressing cassette in site IX-1), and BY4742/GFP_XVI-1 (GFP expressing cassette in site XVI-1) were used in this work. Yeast cells were grown in liquid rich YPD medium or on YPD agar plates at 30 °C. The nonselective YPD medium contained 10 g/L yeast extract (Thermo Fisher (Waltham, MA, USA), #211929), 20 g/L peptone (Thermo Fisher, #211677), and 20 g/L D-(+)-glucose (Merck (Rahway, NJ, USA), #108342). For selection of the Cas9-expressing plasmid, the medium was supplemented with G418 (200 mg/L, InvivoGen (San Diego, CA, USA), #108321-42-2). For selection of plasmids expressing sgRNA, the medium was supplemented with hygromycin B (300 mg/L, InvivoGen, #31282-04-9) and/or phleomycin (30 mg/L, InvivoGen, #11006-33-0) and/or nourseothricin (100 mg/L, Jena Bioscience (Jena, Germany), AB-101-10ML). All cloning was carried out using *Escherichia coli* strain DH5alpha. *E. coli* transformants were grown in LB medium (10 g/L tryptone (Dia-M, RTR.0500), 5 g/L yeast extract (ThermoFisher, #211929), and 10 g/L NaCl (Dia-M, #3436.5000); pH 7.0) containing ampicillin (100 mg/L, Sigma–Aldrich, A1593). Solid media were obtained by the addition of 20 g/L agar (Dia-M, #1923.5000).

### 2.2. Molecular Biological Techniques

PCR amplification of fragments for genomic integration and cloning was performed using Q5^®^ High-Fidelity DNA Polymerase (New England Biolabs (Ipswich, MA, USA), M0491). DNA fragments were purified from the PCR mix using a Cleanup Standard Kit (Evrogen (Moscow, Russia), BC022). PCR for checking colonies was carried out using Taq DNA Polymerase (ThermoFisher, EP0401). Total DNA of yeast strains was isolated using a standard method [13]. Plasmids maintained in *E. coli* were isolated using the Monarch^®^ Plasmid Miniprep Kit (New England Biolabs, T1010).

### 2.3. Plasmids

All plasmids used in this work are listed in Table 1. The Cas9 plasmid pCfB2312 was kindly provided by Dr. Irina Borodina (Technical University of Denmark, Denmark) as a part of the “EasyClone-MarkerFree” vector toolkit [14]. The sgRNA plasmids pCfB9336, pCfB9341, and pCfB9344 were constructed previously [15]. Each sgRNA expression cassette included the *SNR52* promoter, the sgRNA sequence, and the *SUP4* terminator [4]. The plasmids pCfB9341-hphMX and pCfB9344-bleMX were constructed by recombination in vivo [16] as follows. The plasmids pCfB9341 and pCfB9344 were digested by *Sma*I (Thermo Fisher, ER0662). The open-reading frames of hygromycin and phleomycin resistance genes were PCR-amplified using the plasmids pAG26 and pUG66 as the templates and pairs of primers, 1 and 2, and 3 and 4, respectively (Table 2). All resulting fragments were purified from the reaction mix. Then, the linearized vector and the PCR-produced open-reading frames were co-transformed into *S. cerevisiae* with selection on the appropriate antibiotics. Transformants carrying the correctly assembled plasmids were identified by PCR (primers 5 and 6 were used for pCfB9341-hphMX, primers 7 and 8 were used for pCfB9344-bleMX); the total DNA from these transformants was used for transformation of *E. coli*. Then, plasmid DNA was isolated from the *E. coli* transformants. Finally, correct assembly was confirmed by sequence analysis. The plasmid pyEGFP harboring yeast-enhanced green fluorescent protein (yEGFP) [17] under the control of the *TPI1* promoter (*TPI1p)* and *CYC1* terminator (*CYC1t)* was kindly provided by Dmitry Abashkin (Ajinomoto-Genetika Research Institute, Russian Federation).

### 2.4. Preparation of dDNA for Integration

The integration sites used in this work are described in [15]. The dDNAs with 70 bp flanks for recombination were prepared by PCR using the plasmid pyEGFP as a template and primers containing 70 bp sequences homologous to the flanks of integration sites at their 5′-ends (Table 2). Primers 9 and 10 were used for the synthesis of fragments for integration into site II-1, 11, and 12 for integration into site IX-1, and 13 and 14 for integration into site XVI-1. The dDNAs with 500 bp sequences for recombination were prepared by PCR using the chromosomal DNA of strains with the model cassette integrated into sites II-1, IX-1, and XVI-1 as a template. The pairs of primers 15 and 16, 17 and 18, and 19 and 20 were used for this purpose. The 0.5 kb DNA fragments flanking the integration sites (which were used to improve the efficiency of integration of dDNAs with 70 bp flanks) were amplified by PCR using chromosomal DNA of strain BY4742 as a template. Primers 15 and 21 and primers 16 and 24, were used to produce the upstream and downstream flanks of site II-1, respectively; primers 17 and 22 and primers 18 and 25 were used to produce the upstream and downstream flanks of site IX-1; and primers 19 and 23 and primers 20 and 2 were used to produce the upstream and downstream flanks of site XVI-1. All fragments were purified from the PCR mix.

### 2.5. Transformation Protocol

In all cases, 0.3 μg of each plasmid and approximately 1 μg of each dDNA fragment were used. In the case of “added arms” (the 0.5 kb DNA fragments homologous to the flanks of integration sites and not linked to but overlapping with fragments subjected to integration), 0.3 μg of each 0.5 kb fragment was added (the amount equimolar to dDNA with the short arms).

The lithium acetate transformation method is described in [20].

**Figure 3:**
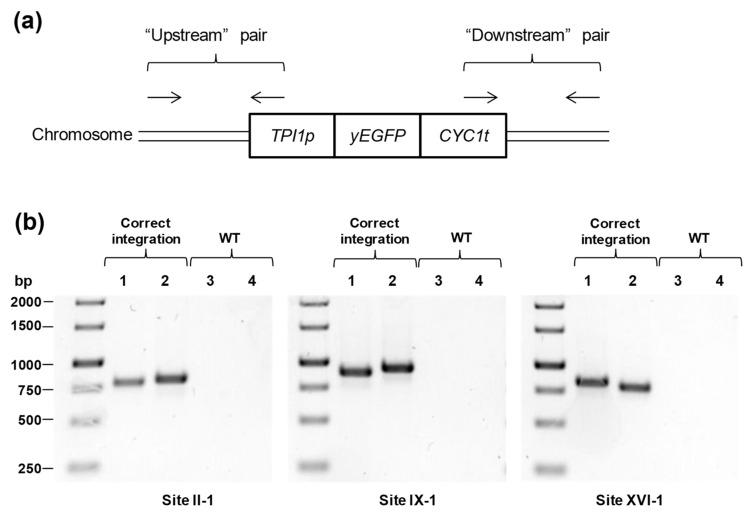
Verification of the integration of the cassettes into target sites. (**a**) Scheme for annealing of the primers. (**b**) Gel electrophoresis of the PCR products. 1, 3—“Upstream” primer pairs. 2, 4—“Downstream” primer pairs. WT—BY4742 cells were used as templates (the negative control).

For electroporation of yeast, the protocol described in [20] was used with minor modifications. YPD medium (10 mL) was inoculated with 0.1 mL of overnight *S. cerevisiae* culture and shaken at 30 °C and 240 rpm until the OD_600_ reached 0.8–1.0. Yeast cells were harvested by centrifugation at room temperature and 6000 rcf for 1 min. The supernatant was discarded, and the cell pellet was washed once with 1 mL of Milli-Q water. Then, the cells were resuspended in 1 mL of LiTED buffer (0.2 M LiAc, pH 7.5; 10 mM Tris*HCl, pH 7.5; 1 mM EDTA; 10 mM DTT) and gently shaken at 30 °C for 1 h. Then, manipulations were carried out in an ice bath. The cells were washed once with 1 mL of cold 1 M sorbitol and once with 1 mL of cold electroporation buffer (1 M sorbitol/1 mM CaCl_2_). Then, the cells were resuspended in 50 μL of the same buffer, and DNA fragments were added to the cells. The resulting mix was placed in a cold electroporation cuvette (Bio-Rad, #1652086) and electroporated on a MicroPulser Electroporator #165-2100 (Bio-Rad, USA) using the “Sc2” program (1.5 kV, 10 μF, time constant of ~5 msec). Immediately after electroporation, cells were suspended in 1 mL of a room-temperature 1:1 mix of 1 M sorbitol and nonselective YPD medium and shaken at 30 °C and 240 rpm for 2 h (when antibiotic markers were used). Finally, the cells were concentrated by centrifugation and spread over YPD agar plates supplemented with appropriate selective antibiotics. Correct integration at the specific genomic site was verified by colony PCR with “upstream” and “downstream” primer pairs. One of the primers in each pair was annealed to the up- or downstream chromosomal area lying outside the integration region, whereas another primer was annealed to the promoter or termination region of the dDNA cassette (Figure 3). Primers 27 and 28 were used as the “upstream” pair, and primers 29 and 30 were used as the “downstream” pair for integration site II-1. Primers 27 and 31 and primers 29 and 32 were used as the upstream and downstream pairs, respectively, for site IX-1; for site XVI-1, primers 27 and 33 and primers 29 and 34 were used as the upstream and downstream pairs, respectively, for site XVI-1.

## 3. Results and Discussion

Most protocols for CRISPR/Cas9-mediated editing of the *S. cerevisiae* genome include the creation of an intermediate strain containing the Cas9 expression system [4,5,6,7]. In some cases, the creation of such a strain may be undesirable; for example, in the case of comparing a large number of strains or in the case of a heavily modified strain, each step of transformation of which is associated with the risk of losing the previous modifications. However, creation of such a strain is not strictly necessary; in some protocols, a plasmid containing the Cas9 expression cassette and the sgRNA expression cassettes was created [8,9]. Assembling such a plasmid for multiple editing is a rather time-consuming procedure, since the new sgRNA expression cassettes are usually prepared and tested as single plasmids (sgRNA plasmids). On the other hand, a high efficiency of triple deletion was achieved when the recipient strain constitutively expressing Cas9 was transformed with three differently marked sgRNA plasmids [6]. Thus, there appear to be no fundamental restrictions on the design of an experiment in which a Cas9-expressing plasmid is introduced into cells together with several sgRNA plasmids. This design would be a more flexible and faster method than the assembly of a single plasmid containing several gRNA expression cassettes and a cassette expressing Cas9. To verify this supposition, we attempted to integrate the model expression cassettes into three sites by means of transformation of a recipient strain with three differently marked sgRNA plasmids, Cas9 expression plasmid bearing the fourth marker, and three dDNAs (GFP expression cassette surrounded with 0.5 kb flanks for recombination with the target loci). The common protocol of lithium acetate transformation [20] was used. The efficiency of simultaneous integration into three loci in this experiment was somewhat lower than when the strain bearing the Cas9 expression plasmid was used as a recipient but remained quite high (75% versus 90%, respectively). However, the number of transformants obtained in this experiment was extremely low (1–3). To increase the transformation efficiency, we applied electroporation as a transformation method. This increased the number of transformants by more than one order of magnitude, while maintaining the same efficiency of simultaneous integration into three loci (Table 3). Thus, simultaneous editing of several loci by co-transformation of a recipient strain with Cas9 expression plasmid and several sgRNA plasmids is possible, and the key point for such an experimental design is the transformation protocol that ensures high transformation efficiency.

Most protocols for CRISPR/Cas9-mediated multiple integration into the *S. cerevisiae* genome use dDNAs with 0.5 kb flanks for recombination with target loci. The addition of these sequences to the integrated DNA fragments is an additional step that also slows the process of genome editing. The high efficiency of triple integration raised the question of how necessary it is to surround the integrated DNA with 0.5 kb flanks. The minimal length of the flanks sufficient to provide integration of the DNA fragment via HR for *S. cerevisiae* is approximately 30 bp [11]. Such sequences can be easily added by PCR [12], and this approach is widely used to manipulate the genes of *S. cerevisiae*. However, elongation of these regions increases the integration efficiency [21]. To address the question of whether dDNA with short flanks can be used for multiple integration when the recipient strain is transformed simultaneously with Cas9 and sgRNA plasmids, we attempted to integrate the model expression cassette into two and three loci. For this, we transformed a recipient strain with differently marked Cas9 and sgRNA plasmids and dDNAs with 70 bp flanks for recombination. We found that integration into two sites occurred with relatively high frequency (45%); however, integration of three cassettes occurred with a frequency of only 6% (Table 4). Thus, adding long arms to integrated DNA is not necessary in the case of integration of two fragments, but is desirable in the case of triple editing.

Long arms for recombination do not have to be covalently linked to the DNA to be integrated, but instead may have short overlapping regions with its ends. This approach was used for traditional genetic manipulations [22,23,24] and for CRISPR/Cas9-mediated manipulations [7,25]. We tested whether this approach can improve the efficiency of triple integration in the case of simultaneous transformation by Cas9 and sgRNA plasmids. As expected, the addition of such arms overlapping the cassettes by 70 bp increased the efficiency of triple integration and it reached 24% (Table 4).

## 4. Conclusions

In this work, we have demonstrated that it is possible to simultaneously skip all preparatory steps used in common protocols for CRISPR/Cas9-mediated multiple genome editing (the construction of an intermediate Cas9-expressing strain, the assembly of a plasmid bearing several sgRNA expression cassettes, and the surrounding integrated DNA fragments with long flanks for recombination with target loci) and integrate at least three dDNA fragments into three loci in one transformation step (Figure 2a). Skipping the creation of an intermediate strain expressing Cas9 only slightly reduced the efficiency of integration; however, it decreased the yield of transformants and therefore required an efficient method of transformation; we used electroporation for this purpose. The shortening of arms for recombination from 0.5 kb to 70 bp had a strong negative effect on the frequency of integration into target loci, but this frequency remained sufficient for integration of two or three DNA fragments. The efficiency of integration of the fragments with short arms can be improved by the addition of long DNA fragments that are homologous to the flanks of the integration sites and not linked to but overlapping with fragments being integrated (Figure 2b).

We believe that the modifications we have tested can be used separately or in combination to accelerate many types of experiments with *S. cerevisiae*, such as evaluation of the overall effect of several genetic modifications, for modification of a large number of strains, or for experiments with heavily modified strains.

## Figures and Tables

**Table 1:** List of plasmids used in this work.

Plasmid	Description	Reference
**Plasmids for marker amplification**		
pAG26	hphMX	[18]
pUG66	bleMX	[19]
**Cas9-carrying plasmid**		
pCfB2312	CEN/ARS, TEF1p-Cas9-CYC1t, kanMX	[14]
**sgRNA-expressing plasmids**		
pCfB9336	2μ, sgRNA II-1, natMX	[15]
pCfB9341	2μ, sgRNA IX-1, natMX	
pCfB9344	2μ, sgRNA XVI-1, natMX	
pCfB9341-hphMX	2μ, sgRNA IX-1, hphMX	This work
pCfB9344-bleMX	2μ, sgRNA XVI-1, bleMX	This work
**Plasmid for dDNA amplification**		
pyEGFP	TPI1p–yEGFP–CYC1t	Lab collection

hphMX—dominant drug resistance cassette providing hygromycin B resistance; bleMX—dominant drug resistance cassette providing phleomycin resistance; kanMX—dominant drug resistance cassette providing G418 resistance; natMX—dominant drug resistance cassette providing nourseothricin resistance.

**Table 2:** List of primers used in this work.

Primer	Sequence (5′ → 3′)	Application
**Primers for replacement of selective markers in plasmids pCfB9341 and pCfB9344**		
1	*cttgctaggatacagttctcacatcacatccgaacataaacaacc*atgggtaaaaagcctgaactc	For amplification of the ORF of hygromycin resistance gene (*hph*)
2	*caaatgacaagttcttgaaaacaagaatctttttattgtcagtactga*ttattcctttgccctcggac	
3	*cttgctaggatacagttctcacatcacatccgaacataaacaacc*atggccgaccaagcgacg	For amplification of the ORF of phleomycin resistance gene (*ble*)
4	*caaatgacaagttcttgaaaacaagaatctttttattgtcagtactga*tcatgagatgcctgcaagcaattc	
5	gatggctgtgtagaagtactcgc	PCR screening for replacement of selective marker with *hph*
6	ctctgtctttcatttaagatgatcat	
7	cgcctgatacagaacgaattgc	PCR screening for replacement of selective marker with *ble*
8	tctcggtaacttgaatggtg	
**Primers for amplification of dDNA fragments containing 70 bp recombination flanks**		
9	*tctttactttgttttaagtagtctttccatctcccttcaacggaaacaagtgcacgcaacatgttatttt*cggatatttaacttacttagaataatgcc	Amplification of dDNA fragment for site II-1
10	*gcaaccgggtagggtgtcttctttgtaaagtttgtctaatgttggagaatggttgccacccggggaagaa*cttcgagcgtcccaaaac	
11	*ctgtttaggaaataaagaagagaaacgtcacctaaaaagtttaaattgaagatgtcgttctatctcgcga*cggatatttaacttacttagaataatgcc	Amplification of dDNA fragment for site IX-1
12	*ccacgctaaccttaatgtgcctagatatcatgggttatttaatgacaagtccgctagtgtcatcaaccca*cttcgagcgtcccaaaac	
13	*tataagaaagtaaacgcaaaagataggctgactgccttcattcgactaggaggtgaggcgacatatttgt*cggatatttaacttacttagaataatgcc	Amplification of dDNA fragment for site XVI-1
14	*ataaaatgggtagaaaaagcatcgttgctgcagtgtacaatcaagctcacccgagccatccacctctcta*cttcgagcgtcccaaaac	
**Primers for amplification of dDNA fragments containing 500 bp recombination flanks**		
15	gttcacagttactcttttagaactcc	Amplification of dDNA fragment for site II-1
16	atgccgtgatatgaacaaacac	
17	ctgtgatcttctaagataaaaaggc	Amplification of dDNA fragment for site IX-1
18	agaaaactacccgtagaatacatattc	
19	gaagattgcaaactcaagtctac	Amplification of dDNA fragment for site XVI-1
20	gttcagtagcaaagtattgtcg	
**Primers for amplification of 500 bp flanks for recombination with desired sites**		
21	aaaataacatgttgcgtgcac	Amplification of 500 bp flank for “upstream” region of site II-1. Used with 15
22	tcgcgagatagaacgacatcttc	Amplification of 500 bp flank for “upstream” region of site IX-1. Used with 17
23	acaaatatgtcgcctcacctc	Amplification of 500 bp flank for “upstream” region of site XVI-1. Used with 19
24	ttcttccccgggtggcaa	Amplification of 500 bp flank for “downstream” region of site II-1. Used with 16
25	tgggttgatgacactagcgg	Amplification of 500 bp flank for “downstream” region of site IX-1. Used with 18
26	tagagaggtggatggctcg	Amplification of 500 bp flank for “downstream” region of site XVI-1. Used with 20
**Primers for testing integration into desired sites**		
27	gctgaaaagtcttagaacgggta	Checking of “upstream” region of dDNA fragment integrated into site II-1
28	tccttatccattcagcttctc	
29	ccgctctaaccgaaaaggaagg	Checking of “downstream” region of dDNA fragment integrated into site II-1
30	accctttttttccattccctc	
31	acagtgagagagtggcat	Checking of “upstream” region of dDNA fragment integrated into site IX-1. Used with 27
32	acttaccaaggtgctgct	Checking of “downstream” region of dDNA fragment integrated into site IX-1. Used with 29
33	acaagagacgaaaaggaccc	Checking of “upstream” region of dDNA fragment integrated into site XVI-1. Used with 27
34	cacttgctttgctcactactc	Checking of “downstream” region of dDNA fragment integrated into site XVI-1. Used with 29

The recombination flanks included in the primers are shown in italics.

**Table 3:** The influence of skipping the creation of an intermediate Cas9-expressing strain on the efficiency of integration into three loci.

Recipient Strain	Transforming Plasmids	Type of Transformation	Number of Transformants	Integration in 3 loci, %
with pCas9	3 sgRNA	LiAc	9 ± 5	90 ± 17
without pCas9	3 sgRNA, pCas9		3 ± 1	75 ± 25
with pCas9	3 sgRNA	Electroporation	240 ± 57	95 ± 2
without pCas9	3 sgRNA, pCas9		46 ± 8	77 ± 9

The integration efficiency shows the ratio of the number of transformants with integration of the cassette into three loci to the total number of transformants. Values represent the mean ± standard deviation from three independent experiments.

**Table 4:** The efficiency of integration of dDNAs with short arms for recombination.

Transforming Plasmids	Added Arms	Proportion of Transformants with the Correct Integration, %	Proportion of Transformants with the Incomplete Integration, %	
			Into 1 Site	Into 2 Sites
2 sgRNA, pCas9 (integration into 2 sites)	no	45 ± 2	14 ± 7	-
3 sgRNA, pCas9 (integration into 3 sites)	no	6 ± 3	2 ± 2	0
3 sgRNA, pCas9 (integration into 3 sites)	yes	24 ± 5	0	0

As “added arms”, the 0.5 kb DNA fragments homologous to the flanks of integration sites and not linked to but overlapping with fragments subjected to integration are denoted. Integration sites II-1 and XVI-1 were used for double integration, and II-1, IX-1, and XVI-1 were used for triple integration. The integration efficiency shows the ratio of the number of transformants with integration of the cassette into target loci to the total number of transformants. Values represent the mean ± standard deviation from three independent experiments.

## Data Availability

Data is contained within the article.
